# Giant cell arteritis in Finland from 2010 to 2020: incidence, developing diagnostic methods and disease presentation

**DOI:** 10.1093/rap/rkaf055

**Published:** 2025-05-15

**Authors:** Lauri Kivitalo, Kirsi Taimen, Tuulikki Sokka-Isler, Anne Kerola, Joonas Rautavaara, Laura Pirilä, Markku Kauppi, Joel Malila, Laura Haara, Laura Ryyppö, Taina Kotijärvi, Panu Saarenketo, Hannu Saarivaara, Juho Siltanen, Mika Helminen, Jarno Rutanen, Pia Isomäki

**Affiliations:** Faculty of Medicine and Health Technology, Tampere University, Tampere, Finland; Department of Internal Medicine, Seinäjoki Central Hospital, Wellbeing Services County of South Ostrobothnia, Seinäjoki, Finland; Centre for Rheumatology and Clinical Immunology, Department of Medicine, Turku University Hospital, Turku, Finland; Faculty of Medicine, University of Turku, Turku, Finland; Faculty of Health Sciences, University of Eastern Finland, Kuopio, Finland; Unit of Rheumatology, Hospital Nova, Wellbeing Services County of Central Finland, Jyväskylä, Finland; Department of Internal Medicine, Päijät-Häme Central Hospital, Wellbeing Services County of Päijät-Häme, Lahti, Finland; Institute for Molecular Medicine Finland, Helsinki Institute of Life Science, University of Helsinki, Helsinki, Finland; Department of Internal Medicine, Päijät-Häme Central Hospital, Wellbeing Services County of Päijät-Häme, Lahti, Finland; Centre for Rheumatology and Clinical Immunology, Department of Medicine, Turku University Hospital, Turku, Finland; Faculty of Medicine, University of Turku, Turku, Finland; Department of Internal Medicine, Päijät-Häme Central Hospital, Wellbeing Services County of Päijät-Häme, Lahti, Finland; Faculty of Medicine, Clinicum, University of Helsinki, Helsinki, Finland; Faculty of Medicine and Health Technology, Tampere University, Tampere, Finland; Faculty of Medicine and Health Technology, Tampere University, Tampere, Finland; Centre for Rheumatology and Clinical Immunology, Department of Medicine, Turku University Hospital, Turku, Finland; Centre for Rheumatology and Clinical Immunology, Department of Medicine, Turku University Hospital, Turku, Finland; Unit of Rheumatology, Hospital Nova, Wellbeing Services County of Central Finland, Jyväskylä, Finland; Unit of Rheumatology, Hospital Nova, Wellbeing Services County of Central Finland, Jyväskylä, Finland; Unit of Rheumatology, Hospital Nova, Wellbeing Services County of Central Finland, Jyväskylä, Finland; Tays Research Services, Wellbeing Services County of Pirkanmaa, Tampere, Finland; Faculty of Social Sciences, Tampere University, Health Sciences, Tampere, Finland; Faculty of Social Sciences, Tampere University, Health Sciences, Tampere, Finland; Centre for Rheumatic Diseases, Tampere University Hospital, Tampere, Finland; Faculty of Medicine and Health Technology, Tampere University, Tampere, Finland; Centre for Rheumatic Diseases, Tampere University Hospital, Tampere, Finland

**Keywords:** giant cell arteritis, vasculitis, incidence, diagnosis, ultrasound, biopsy, phenotype

## Abstract

**Objectives:**

To study the annual incidence, diagnostic methods used and clinical presentation of giant cell arteritis (GCA) over time in Finland.

**Methods:**

Newly diagnosed GCA patients from 2010 to 2020 were retrospectively identified from four healthcare districts in Finland. Medical records were reviewed and data on incidence, diagnostic methods, phenotype [cranial *vs* large vessel (LV)-GCA] and clinical presentation were analysed.

**Results:**

We identified 602 newly diagnosed GCA patients. The annual incidence was 9.0 cases/100 000 persons (95% CI 8.3, 9.7) ≥50 years of age and was significantly higher in the period 2016–2020 compared with the period 2010–2015 [11.3 (95% CI 10.1, 12.5) *vs* 7.0 (95% CI 6.2, 7.9), *P* < 0.001]. Imaging- or biopsy-confirmed diagnosis was recorded in 75% of GCA patients, while 25% had a clinical diagnosis. The proportion of imaging- or biopsy-confirmed diagnoses increased over time [64.7% (2010–2015) *vs* 82.2% (2016–2020)] while that of clinical diagnoses decreased. The use of imaging methods increased while the use of temporal artery biopsies decreased between the two time periods. LV-GCA was discovered more often in the period 2016–2020 when compared with 2010–2015 (34.0% *vs* 19.3% of patients).

**Conclusion:**

The incidence of GCA increased during the study period, as well as the proportion of imaging- or biopsy-confirmed diagnoses, probably due to more frequent use of advanced imaging methods. Additionally, patients with LV-GCA were more commonly identified.

## Graphical abstract

**Figure:**
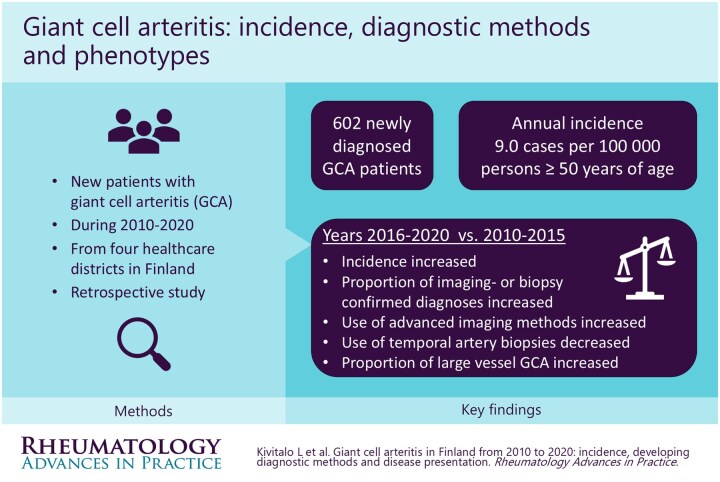

Key messages

- This large cohort study indicates an increase in the incidence of GCA during the 2010s in Finland.
- During 2010–2020, the proportion of biopsy- and imaging-confirmed diagnoses and LV-GCA increased.
- Imaging methods were used more frequently, while the need of temporal artery biopsies decreased.

## Introduction

GCA is a systemic vasculitis in people ≥50 years of age. It presents as cranial arteritis, e.g. temporal arteritis, and/or as large vessel arteritis (LV-GCA) involving the aorta and/or its branches [1]. Typical complaints of GCA are cranial symptoms (e.g. temporal headache, jaw claudication, visual loss), polymyalgic symptoms (morning stiffness and pain in the proximal muscles) and unspecific general symptoms (e.g. fever, fatigue, weight loss). The ESR and CRP are usually elevated. The new international ACR/EULAR 2022 classification criteria have been recently published to replace the old ACR 1990 classification criteria, and the new criteria also include the non-cranial phenotype of GCA [2, 3].

Advanced imaging methods have become available in recent years to improve the diagnostics of GCA [4, 5]. US examination of temporal and other arteries has been introduced as the primarily recommended investigation method. In addition, the availability of ^18^F-fluorodeoxyglucose PET-CT imaging has increased. CT and MRI scans may also visualize arteritis of medium size and large vessels.

In previous studies, the incidence of GCA has varied considerably and many studies involve patients diagnosed in the era when modern diagnostic methods were not available [6]. In register studies from Nordic countries, high incidence rates have been reported: 16.7 cases/100 000 persons ≥50 years of age in Norway from 1972 to 2012 [7], 22.2 in Denmark from 1996 to 2018 [8] and 13.3 in Sweden from 1997 to 2019 [9]. In other parts of Europe, lower incidence rates have been reported. A meta-analysis published in 2021 reported a GCA incidence of 7.26 in Europe [10]. In recent studies from the UK and Spain the incidence of GCA in the 2010s was 9.8 and 7.4, respectively. In the UK, but not in Spain, there was an increase in the incidence during the study period [11, 12].

Given the variability in the reported incidence of GCA between different studies and the recent advances in diagnostic methods, we collected and verified from medical records a large cohort of GCA patients to evaluate the annual incidence of GCA, the diagnostic methods used and disease presentation over 11 years in Finland.

## Methods

This retrospective multicentre study was conducted in four healthcare districts (Pirkanmaa, Southwest Finland, Central Finland and Päijät-Häme) that comprise >25% of Finland’s population and include 553 998 (year 2010) to 616 774 (year 2020) persons ≥50 years of age (Supplementary Fig. S1, available at *Rheumatology Advances in Practice* online). Population data were obtained from the Finnish Institute for Health and Welfare (THL).

### Patients

New GCA patients diagnosed between 1 January 2010 and 31 December 2020 were retrospectively identified based on the hospital inpatient or outpatient diagnosis codes. In the first phase, patients were screened based on International Classification of Diseases, Tenth Revision (ICD-10) codes for GCA (M31.5 or M31.6) and codes for Takayasu arteritis (M31.4) and unspecified arteritis (I77.6) to cover GCA inaccurately recorded with these codes. To exclude occasional false ICD codes, the diagnosis code was required to occur in at least three patient contacts. In the second phase, the medical records were reviewed in each rheumatology unit by a rheumatologist or a registrar with experience in GCA. The diagnosis of GCA was verified based on temporal artery biopsy (TAB) or imaging findings or according to clinical judgement as defined by the Chapel Hill Consensus Conference [13]. The follow-up time from diagnosis to data collection was at least 1 year, which ensured that the diagnoses were correct.

The following exclusion criteria were used: diagnosis was considered to be other than GCA (e.g. isolated PMR, infection, malignancy or IgG4-related disease explaining GCA-like symptoms), the cause of symptoms was indefinite, GCA diagnosis was established before 2010 or GCA diagnosis was done in another hospital district.

### Data collection and permissions

Pseudonymized data were collected from electronic health records of participating hospitals into an electronic data collection platform (REDCap). The clinical data at the diagnostic phase and during the follow-up were collected according to EULAR recommendations on the minimum core dataset to be collected in GCA registries and databases [14]. In addition, we collected information on symptom duration and diagnosis delay. Imaging methods recorded were US, PET-CT, CT and MRI [13]. Data on GCA phenotypes was also collected. Cranial GCA is GCA confirmed by TAB or temporal artery US examination. LV-GCA is GCA with vasculitis in the aorta and/or its branches confirmed by imaging (PET-CT, CT, US or MRI).

The medical director of each participating hospital district approved a research permit and a joint registry permit and the data are stored in the Pirkanmaa healthcare district database in pseudonymized form. Since this is a retrospective registry study, permission from the ethics committee or patients was not required according to Finnish law.

### Statistics

The annual incidence of GCA was reported as the number of new cases per 100 000 persons ≥50 years of age with 95% CIs based on normal approximation (Wald method). Individuals <50 years of age were excluded from incidence calculations. Annual incidence and categorical values between two time periods were compared using the chi-squared test. Age and inflammatory parameters between two periods were compared with the Student’s *t*-test and diagnosis delay with the Mann–Whitney U test. The chi-squared test was used to compare incidences and categorical values and analysis of variance was used to compare continuous variables (age, inflammatory parameters and diagnosis delay) between research units.

The data were analysed using SPSS version 29.0.1.0 (IBM, Armonk, NY, USA). *P*-values <0.05 were considered statistically significant.

## Results

**Figure 1.**
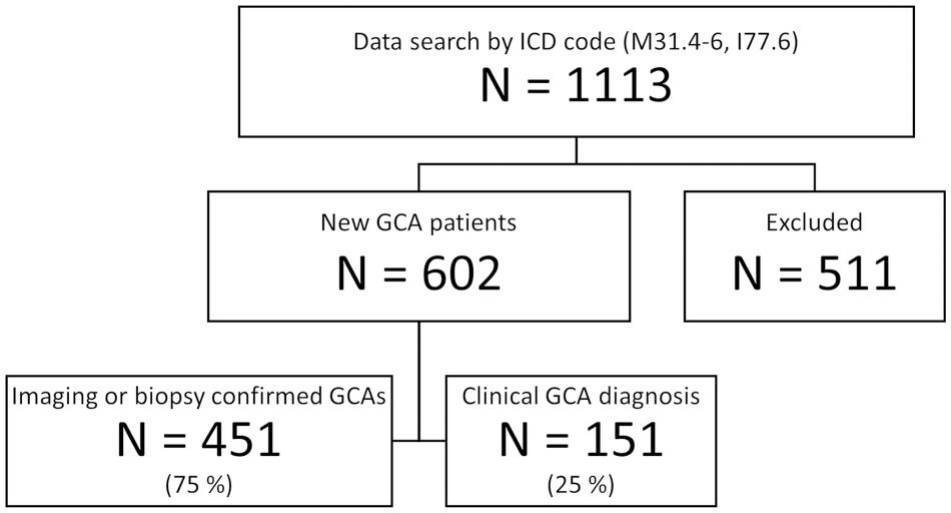
New GCA diagnoses from four healthcare districts in Finland from 2010 to 2020. Patients were excluded from the study if the diagnosis was considered to be other than GCA, the cause of symptoms was indefinite or the GCA diagnosis was before 2010 or in another healthcare district

A data search for specific diagnosis codes identified 1113 patients meeting the inclusion criteria (Fig. 1). Of these, 511 patients were excluded due to inaccurate diagnosis of GCA or due to a GCA diagnosis before 2010 or in another hospital district. We thus identified 602 new GCA patients diagnosed during 2010–2020, of which 451 (75%) had an imaging- or biopsy-verified diagnosis and 151 (25%) had a clinical diagnosis. The proportion of females was 68.4% and the mean age at diagnosis was 72.1 years (s.d. 8.4). One 49-year-old patient with biopsy-confirmed GCA was excluded from the incidence calculations.

### Incidence

**Figure 2.**
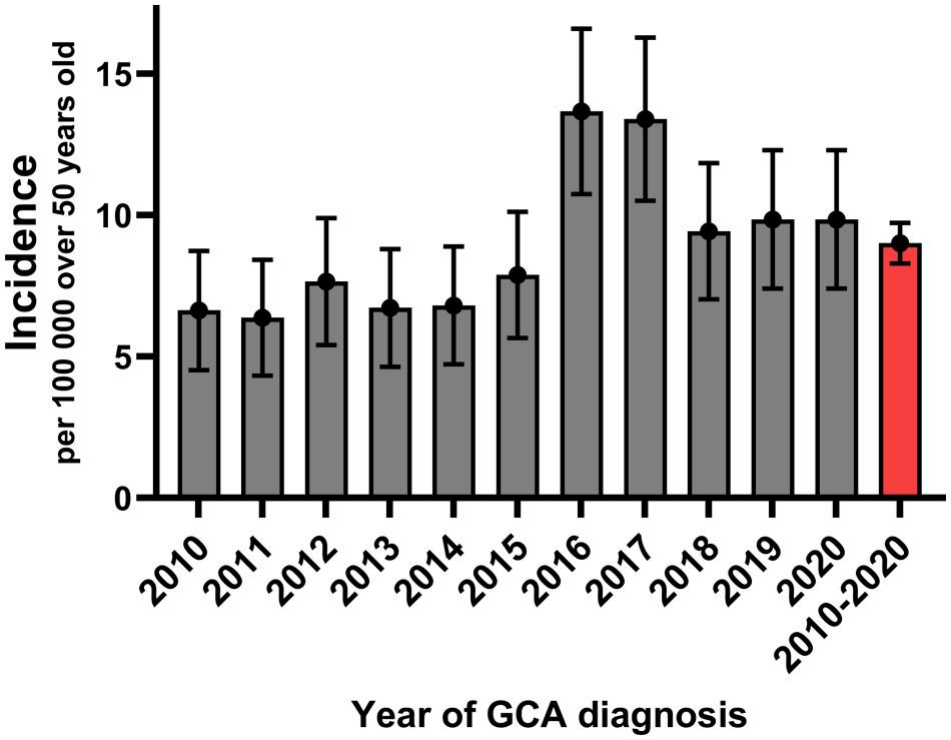
Incidence of GCA in Finland from 2010 to 2020. Annual incidence and 95% CIs are presented

The annual incidence of GCA during 2010–2020 was 9.0 cases per 100 000 persons (95% CI 8.3, 9.7) ≥50 years of age (Fig. 2). There was no significant difference in the incidence between the four healthcare districts of the study (*P* = 0.751; Supplementary Fig. S2, available at *Rheumatology Advances in Practice* online).

The incidence among females was 11.4 (95% CI 10.3, 12.6) and among males was 6.2 (95% CI 5.2, 7.1). The incidences by age group were 50–59 years 2.3 (95% CI 1.7, 3.0), 60–69 years 9.3 (9%5% CI 8.0, 10.6), 70–79 years 15.8 (95% CI 13.7, 17.8), 80–89 years 15.0 (95% CI 12.2, 17.8) and 90–99 years 2.8 (95% CI 0.1, 5.5).

The annual incidence of GCA was rather stable during 2010–2015, increased markedly in 2016 and remained at a higher level during the following years. The annual incidence of GCA was significantly higher in the period 2016–2020 compared with the period 2010–2015 [11.3 (95% CI 10.1, 12.5) *vs* 7.0 (95% CI 6.2, 7.9), *P* < 0.001; Fig. 2]. The increase in incidence was observed in all four study sites (Supplementary Fig. S2, available at *Rheumatology Advances in Practice* online).

The incidence of GCA was higher in 2016 and 2017 than in other years. There was no significant monthly or seasonal variation in the diagnosis of GCA or in the start of GCA symptoms (Supplementary Fig. S3, available at *Rheumatology Advances in Practice* online).

The median diagnosis delay (time from the onset of GCA symptoms to the initiation of glucocorticoid treatment for GCA) was 28 days [interquartile range (IQR) 14–66] and was comparable between 2010–2015 [28 days (IQR 14–54)] and 2016–2020 [31 days (IQR 14–76)] (*P* = 0.210). In the high-incidence years 2016 and 2017, the diagnosis delay did not differ from the other years (data not shown).

Given the differential annual incidence of GCA in the period 2016–2020 compared with 2010–2015, we decided to compare the diagnostic methods used and the phenotype and clinical presentation of GCA between these two time periods.

### Diagnostic methods

**Figure 3.**
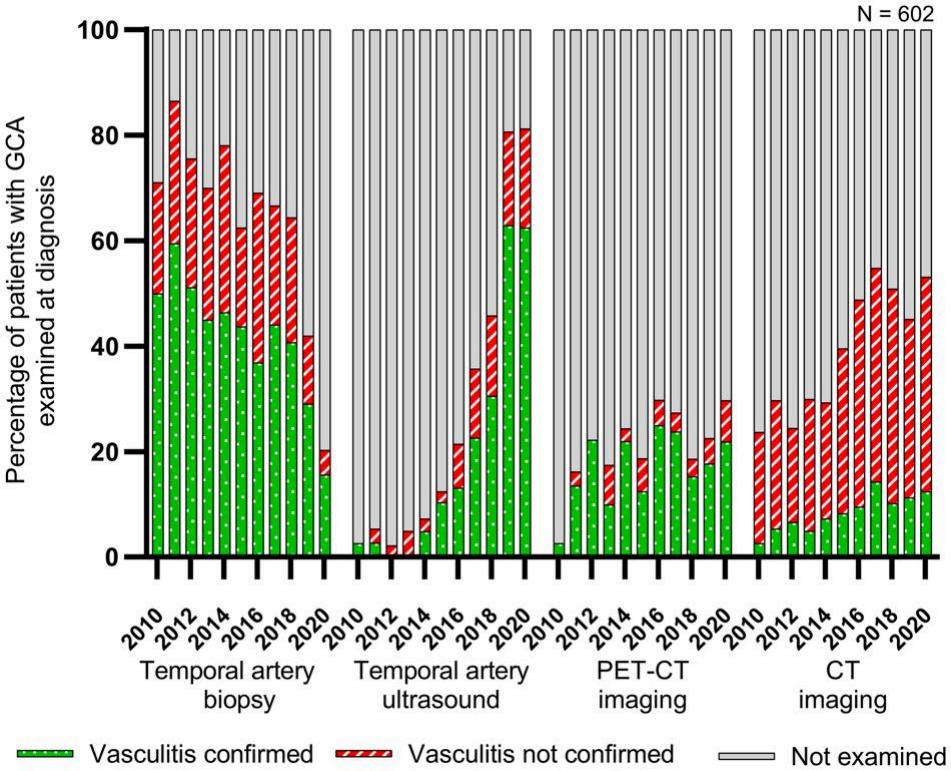
The annual (from 2010 to 2020) percentages of different investigation methods performed at diagnosis for patients with GCA. PET-CT means ^18^F-fluorodeoxyglucose PET-CT. CT means any CT imaging of arteries

In the beginning of the study period, the most common investigation method among patients with GCA was TAB (Fig. 3). During the entire study period, biopsy was performed for 62.1% of patients and it confirmed vasculitis for 40.2% of all GCA patients. However, the proportion of patients in whom TAB was performed significantly decreased from 2010–2015 to 2016–2020 (73.5% *vs* 54.1%, *P* < 0.001). The number of biopsies decreased, especially during 2019 and 2020 (Fig. 3).

Arterial US examinations, performed mainly by rheumatologists, were more frequent towards the end of the study period (Fig. 3). During the entire study period, temporal artery US examination was performed in 31.9% of GCA patients and the examination confirmed vasculitis in 22.6% of all GCA patients. The proportion of patients in whom temporal artery US examination was performed increased significantly over time (6.0% in 2010–2015 *vs* 50.1% in 2016–2020, *P* < 0.001).

PET-CT imaging of large vessels was performed in 22.4% of GCA patients and it confirmed vasculitis in 18.3% of patients (Fig. 3). Any CT imaging (most commonly a body CT for differential diagnosis) was performed in 42.0% of patients and vasculitis was confirmed by CT in 9.3% of cases. The proportion of both PET-CT and CT imaging was significantly higher in 2016–2020 compared with 2010–2015 (PET-CT 26.1% *vs* 17.3%, *P* = 0.011; CT 50.7% *vs* 29.7%, *P* < 0.001).

The use of MRI, extracranial artery US and other investigation methods was less common. US examination of arteries other than the temporal artery confirmed vasculitis in 37 patients (6.1%): axillary artery in 18 (3.0%), carotid artery in 11 (1.8%), subclavian artery in 2 (0.3%) and any other artery in 9 patients (1.5%; Supplementary Table S1, available at *Rheumatology Advances in Practice* online). MRI confirmed vasculitis in 3 patients (0.5%), another biopsy target than the temporal artery (e.g. aorta from surgery) in 3 patients (0.5%) and another imaging method (e.g. conventional angiography) in 2 patients (0.3%; Supplementary Table S1, available at *Rheumatology Advances in Practice* online).

### Phenotypes of GCA

**Figure 4.**
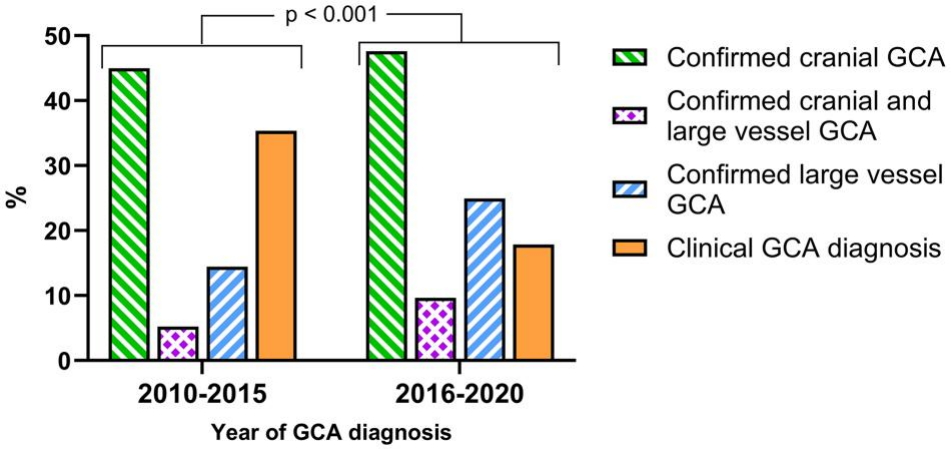
The phenotypes of new GCA patients in 2010–2015 *vs* 2016–2020. Confirmed cranial GCA indicates a vasculitis finding in the TAB and/or temporal artery US examination. Confirmed LV-GCA indicates a vasculitis finding in at least one of the large vessel investigation methods (e.g. PET-CT imaging, CT imaging or large vessel US). It should be noted that the diagnostic examinations performed for each patient were individually selected by the treating physician. *P*-value indicates the difference in the distribution of different phenotypes in 2010–2015 and 2016–2020

The phenotypes of GCA changed significantly between the time periods (*P* < 0.001; Fig. 4). The proportion of diagnoses based on plain clinical judgement decreased over time (35.3% in 2010–2015 *vs* 17.8% in 2016–2020), while the imaging- or biopsy-confirmed diagnoses increased (64.7% in 2010–2015 *vs* 82.2% in 2016–2020). There were no differences in the proportions of clinical diagnoses between the four healthcare districts (Supplementary Fig. S4, available at *Rheumatology Advances in Practice* online).

The proportion of cranial GCA remained unchanged between the two time periods (45.0% *vs* 47.6%; Fig. 4). In contrast, the identification of LV-GCA was higher in the period 2016–2020 compared with 2010–2015 (24.9% *vs* 14.5%). The number of patients with confirmed cranial and LV-GCA was relatively small (5.2% in 2010–2015 *vs* 9.6% in 2016–2020). One study unit used significantly more PET-CT imaging and fewer temporal artery examinations (biopsy and US) and therefore there was a higher proportion of LV-GCA than in other study sites (Supplementary Fig. S4, available at *Rheumatology Advances in Practice* online).

### Fulfilment of classification criteria

The ACR/EULAR 2022 classification criteria were met by 84.1% and the older ACR 1990 classification criteria by 77.7% of the patients with GCA. There was no difference between the two time periods in fulfilling ACR/EULAR 2022 classification criteria (83.9% in 2010–2015 *vs* 84.1% in 2016–2020, *P* = 0.947), whereas the ACR 1990 classification criteria were met less frequently in the latter period (84.3% in 2010–2015 *vs* 73.1% in 2016–2020, *P* = 0.001). Of the GCA patients diagnosed by plain clinical judgement, only 14 (2.3%) did not meet either the ACR/EULAR 2022 or ACR 1990 classification criteria.

### Clinical presentation of GCA

In order to study whether the clinical presentation of GCA changed within the study period, patient demographics, GCA symptoms and clinical findings were also compared between the periods 2010–2015 and 2016–2020. The proportion of males was slightly higher in the latter period but there were no statistically significant differences in patient age, sex distribution or smoking between the two time periods (Table 1).

**Table 1.** Clinical presentation of GCA at diagnosis in the total patient cohort and in the periods 2010–2015 and 2016–2020.

Characteristics	Total (*N* = 602)	2010–2015 (*n* = 249)	2016–2020 (*n* = 353)	*P*-value^a^
Female, %	68	72	66	0.088
Age, years, mean (s.d.)	72 (8)	73 (9)	72 (8)	0.375
Current smoker, %^b^	16	17	16	0.747
Any visual symptom, %	45	44	45	0.833
Permanent visual loss in ≥1 eye, %	8	9	7	0.512
New headache, %	73	78	70	0.023
New temporal headache, %	62	67	59	0.063
Jaw or tongue claudication, %	43	38	47	0.024
Scalp tenderness, %	37	39	36	0.566
Abnormal temporal artery palpation, %^c^	51	58	46	0.005
Proximal muscle pain or stiffness, %	41	41	40	0.779
Fever, %	32	31	34	0.410
Fatigue, %	45	39	50	0.007
Weight loss, %	25	23	26	0.280
Limb claudication, %	5	3	6	0.096
Peripheral pulse abnormality, %	3	2	3	0.483
CRP, mg/l, mean (s.d.)	99 (78)	99 (77)	99 (79)	0.980
ESR, mm/h, mean (s.d.)	75 (31)	77 (31)	74 (31)	0.157
CRP ≥10 mg/l, %	96	94	97	0.125
ESR ≥50 mm/h, %	76	78	74	0.264
CRP <10 and ESR <50, %	4	4	3	0.402

*P*-values indicate differences between the two time periods.

a The chi-squared test was used for categorical variables and the Student’s *t*-test for continuous variables.

b Smoking data available for 555 (92%) patients.

c Tenderness, hardness or decreased pulsation.

Regarding the symptoms and clinical findings of GCA, new headache (70% *vs* 78%, *P* = 0.023) and abnormal temporal artery palpation (46% *vs* 58%, *P* = 0.005) occurred less frequently, whereas jaw or tongue claudication (47% *vs* 38%, *P* = 0.024) and fatigue (50% *vs* 39%, *P* = 0.007) occurred more frequently in the later period compared with the earlier period (Table 1). There was no difference in the prevalence of visual symptoms, permanent visual loss, scalp tenderness, proximal muscle pain or stiffness, fever, weight loss, limb claudication and peripheral pulse abnormality between the time periods. There were no differences in the CRP and ESR values at diagnosis between the two time periods.

## Discussion

In this large cohort study, we demonstrate an increase in the incidence of GCA in Finland during 2010–2020. During this time, the use of diagnostic methods changed, with fewer TABs and more temporal artery US and PET-CT examinations performed towards the end of study period. Concurrently, the proportion of GCA diagnoses based on plain clinical judgement decreased while the proportion of LV-GCA increased.

In recent European studies also from the 2010s, the annual incidences of GCA have been rather comparable to the incidence of 9.0 cases/100 000 persons ≥50 years of age reported in the current study; in the UK, the incidence was 9.8 during 2011–2020 (diagnosis based on ACR/EULAR 2022 classification criteria) and in Spain the incidence was 7.4 during 2013–2019 (diagnosis based on clinical judgement) [11, 12]. The sex- and age-specific incidences in our study were also comparable to those reported in the UK and Spain. In Italy, the incidence was similar at 8.3 during 2005–2016 (diagnosis based on modified GiACTA inclusion criteria) [15]. Interestingly, in the UK cohort the incidence was higher during 2017–2020 than 2011–2016 [11], consistent with the increase in GCA incidence towards the end of the study period reported in our study. In contrast, in a Danish study the incidence remained constant during 1996–2018, and in Swedish TAB-based data from 1997 to 2019, the incidence decreased during 2017–2019 (especially in 2019), probably due to a decrease in biopsies performed to patients with suspected GCA. Therefore, it seems that the inclusion criteria of the studies may significantly influence the temporal trends of the GCA incidence observed [8, 9].

Despite the higher annual incidence of GCA during 2016–2020 observed in our study, the incidence of 11.3 was still lower than that reported in previous studies from other Nordic countries. In a large cohort study from Denmark the incidence of GCA was 22.2 during 1996–2018 (register study by GCA code and glucocorticoid prescription) [8]. The incidence was 16.7 in Norway during 1972–2012, based on the fulfilment of ACR 1990 classification criteria [7]. In a Swedish study based on a positive TAB finding, the incidence was 13.3 during 1997–2019 [9]. High GCA incidences have also been observed in other previous studies from Nordic countries [6, 10]. The contribution of genetic factors to the high incidence of GCA in Nordic countries has been suggested. For example, the variable population prevalence of HLA-DRB1*04 alleles that are associated with GCA might partially explain the difference in GCA incidence between countries [16, 17].

Previous smaller studies from Finland have demonstrated lower incidences of GCA than those reported from other Nordic countries. The incidence of GCA in North Savo area in Finland was 7.5 in 2010 (fulfilment of ACR 1990 criteria) [18] and 9.2 in 1980–1989 (positive TAB finding) [19]. The 9.0 incidence of GCA reported in the current study is in line with previous Finnish cohorts but markedly lower than that reported from other Nordic countries. Whether this reflects variabilities in study methods or actual differences in the occurrence of GCA between Finland and other Nordic countries remains to be shown in future studies. We expect our results report the reliable incidence of GCA, since in our study GCA diagnosis was validated through a review of medical records. It should be noted that if inclusion of patients to our GCA cohort had been done on the basis of diagnosis codes only, this would have resulted in a significant overestimation of GCA incidence.

The reason for the higher incidence of GCA in 2016 and 2017 compared with other years in the current study is unclear. Peak incidence years have also been observed in other studies, suggesting involvement of environmental factors, such as viral infections. In Denmark, a peak incidence of GCA and PMR was observed in connection with *Mycoplasma pneumoniae*, *Chlamydia pneumoniae* and parvovirus B19 epidemics [20]. In a large meta-analysis, prior infections—especially within 1 year or prior herpes zoster infection—were associated with a higher incidence of CGA [21]. Involvement of infections in the onset of GCA might suggest seasonal variation in the incidence. Indeed, in Denmark, GCA appears more in the summer months, and in Sweden and Spain in the spring months [9, 12, 22]. However, we did not observe a seasonal variation in GCA incidence, nor did a recent meta-analysis [21], suggesting that there seems to be no consistent seasonal variation in GCA incidence.

Diagnostic examinations performed for GCA patients changed remarkably during our study. Towards the end of study period, US examination of temporal arteries replaced the TAB, in accordance with EULAR’s recommendation [4]. During the study period, the diagnostic capability of US devices probably also improved due to technical developments, and the rheumatologists’ skills of arterial US examination improved. In a prospective register of data collected in German-speaking countries in 2019–2023, the proportion of patients on whom TABs and imaging methods were performed at diagnosis was at the same level as during the last years of our study [23].

This development in diagnostic methods performed for GCA patients did not influence the proportion of GCA patients with cranial disease, which was comparable during 2010–2015 and 2016–2020. In contrast, the proportion of LV-GCA significantly increased towards the end of the study, most likely due to more common use of PET-CT, CT and US imaging. However, the observed distribution of LV-GCA and cranial disease depends significantly on the investigation methods used at diagnosis (e.g. frequency of temporal artery investigations *vs* PET-CT or CT). It is thought that 80% of patients with GCA will have LV involvement on PET-CT or US if systemically investigated [24, 25]. According to EULAR’s recommendations, when a GCA diagnosis is confirmed, it is unnecessary to do further investigations (e.g. if US confirms temporal arteritis, it is not recommended to search for LV involvement with PET-CT) [4]. This policy was used for most of the patients in the current study, explaining the low number of patients with confirmed cranial and concurrent LV involvement. As diagnostic methods have improved and become more readily available, the proportion of patients diagnosed based on plain clinical judgement has decreased. The proportion of clinical diagnoses in our study (25%) was rather similar to those reported in recent Spanish (25%) and Italian (17%) studies [12, 26].

Due to more frequent use of advanced imaging methods, patients with GCA may be better identified and confirmed. In the past, a larger proportion of GCA patients have possibly gone unrecognized or have been treated for another diagnosis such as PMR or suspected unspecified inflammatory disease. Increased awareness among doctors of the spectrum of GCA disease presentations may also have resulted in increased referral of patients for investigation. Due to advanced and increased imaging, the demonstrated annual incidence of GCA (11.6) during 2016–2020 in the current study is likely to be closer to reality than the annual incidence of the entire study period. Of note, the increased use of imaging did not speed up or delay the diagnostics, since diagnosis delay from the onset of symptoms remained stable during the study period. This may be explained by the fact that the longest delays are likely due to patients not seeking medical consultation promptly and doctors in primary healthcare not recognizing symptoms of GCA.

Our study has several strengths, including a large patient cohort representing >25% of the Finnish population. Medical records were manually reviewed by doctors with experience in GCA and patients were followed up for at least 1 year, making diagnosis of GCA reliable. The fulfilment of the newly published ACR/EULAR 2022 classification criteria was also examined. In addition, the relatively long study period of 11 years dates to the change in diagnostic methods, allowing us to relate the changes in incidence and phenotype of GCA to the development of diagnostic procedures performed.

A limitation of our study is the retrospective design. Since symptoms and clinical findings were not systematically collected, the proportion of patients with certain symptoms and findings may be somewhat underestimated. In addition, our patient cohort contained only GCA cases diagnosed and treated at hospital units, which might have led to underestimation of GCA incidence. However, GCA is hardly ever diagnosed and treated in primary healthcare units or in private clinics in Finland, making significant underestimation of GCA incidence in the current study an unlikely scenario.

In conclusion, our study confirms a significant change in the diagnostic methods of GCA in the 2010s. Concurrently the incidence of GCA increased, as did the proportion of LV-GCA and the proportion of imaging- and biopsy-confirmed GCA cases. It remains to be shown whether the increase in GCA incidence in Finland during the study period is explained solely by advances in diagnostics or whether the incidence has truly increased in recent years. Nevertheless, our results suggest that patients with GCA can today be better identified and modern, non-invasive diagnostic methods should be readily available in clinics diagnosing and treating patients with GCA.

## Supplementary Material

rkaf055_Supplementary_Data

## Data Availability

Anonymized data are available from the authors upon reasonable request.
