# Supplementary material for: Giant cell arteritis in Finland from 2010 to 2020: incidence, developing diagnostic methods and disease presentation
Source: Rheumatol Adv Pract. 2025 May 15;9(2):rkaf055. doi: 10.1093/rap/rkaf055 (PMC12145172; doi:10.1093/rap/rkaf055)
Supplement: rkaf055_Supplementary_Data [file rkaf055_supplementary_data.docx]

Supplementary material

**Supplementary Figure S1.** Annual ≥ 50 years old population in four healthcare districts in the study.

**Supplementary Figure S2.** Annual incidence of GCA in four healthcare districts from 2010 to 2020.

**Supplementary Figure S3.** Monthly incidence of GCA in 2010-2020.

**Supplementary Table S1.** Investigation methods of GCA which confirmed vasculitis at diagnosis. Table presents the number of patients and the percentage of all GCA patients with vasculitis finding in the investigation method performed.

|  | N | % |
| --- | --- | --- |
| Temporal artery biopsy | 242 | 40.2 |
| Temporal artery ultrasound | 136 | 22.6 |
| PET-CT imaging | 110 | 18.3 |
| CT imaging | 56 | 9.3 |
| Large vessel ultrasound | 37 | 6.1 |
| *axillary artery* | *18* | *3.0* |
| *carotid artery* | *11* | *1.8* |
| *subclavian artery* | *2* | *0.3* |
| *other artery* | *9* | *1.5* |
| MRI | 3 | 0.5 |
| Other biopsy target than temporal artery | 3 | 0.5 |
| Other imaging method | 2 | 0.3 |

**Supplementary Figure S4.** The phenotypes of new GCA patient in four healthcare districts**.** P-value indicates the difference in the distribution of different phenotypes in four healthcare districts.
